# Intradialytic Cardiovascular Exercise Training Alters Redox Status, Reduces Inflammation and Improves Physical Performance in Patients with Chronic Kidney Disease

**DOI:** 10.3390/antiox9090868

**Published:** 2020-09-15

**Authors:** Apostolos Sovatzidis, Athanasios Chatzinikolaou, Ioannis G. Fatouros, Stylianos Panagoutsos, Dimitrios Draganidis, Eirini Nikolaidou, Alexandra Avloniti, Yiannis Michailidis, Ioannis Mantzouridis, Alexios Batrakoulis, Ploumis Pasadakis, Vassilis Vargemezis

**Affiliations:** 1 Department of Nephrology, Faculty of Medicine, University Hospital of Alexandroupolis, Democritus University of Thrace, 68100 Alexandroupolis, Greece; spanagou@med.duth.gr; 2 School of Physical Education and Sport Sciences, Democritus University of Thrace, 69100 Komotini, Greece; athchatz.tefaa@gmail.com (A.C.); alavloni@phyed.duth.gr (A.A.); michailidis79@hotmail.com (Y.M.); ioanmatz3@med.duth.gr (I.M.); 3 School of Physical Education and Sport Sciences, University of Thessaly, Karies, 42100 Trikala, Greece; fatouros@otenet.gr (I.G.F.); dimidraganidis@gmail.com (D.D.); alexis_batrakoulis_75@hotmail.com (A.B.); 4 Department of Plastic, Reconstructive and Hand Surgery & Burns ICU, General Hospital of Thessaloniki “G. Papanikolaou”, 57010 Thessaloniki, Greece; eirininikolaidou7@gmail.com

**Keywords:** chronic kidney disease, hemodialysis, exercise, training, oxidative stress, inflammation

## Abstract

Redox status (RS) perturbations and inflammation are fundamental features of chronic kidney disease (CKD) that are substantially exacerbated in end-stage renal disease (ESRD). This study aimed at investigating the efficacy of a 6-month intradialytic exercise training program on RS, inflammation and physical performance in patients with ESRD. Twenty hemodialysis (HD) patients (17 males, three females) were randomly assigned to either an intradialytic training (bedside cycling) group (TR; *n* = 10) or a control group (CON; *n* = 10) for 6 months. Anthropometrics [body mass and height, body mass index (BMI), body composition], physical performance (VO_2peak_), functional capacity [North Staffordshire Royal Infirmary (NSRI) walk test, sit-to-stand test (STS-60)], quality of life (short form-36 (SF-36) as well as RS [thiobarbituric acid reactive substances (TBARS), protein carbonyls (PC), reduced (GSH) and oxidized (GSSG) glutathione, GSH/GSSG, total antioxidant capacity (TAC), catalase activity (CAT)] and high-sensitivity C-reactive protein (hs-CRP) were assessed at baseline and after the 6-month intervention. Peak oxygen consumption (VO_2peak_) increased by 15% only in TR (*p* < 0.01). Performance in NSRI, STS-60 and SF-36 improved by 4–13% only in TR (*p* < 0.01). Exercise training reduced TBARS (by 28%), PC (by 31%) and hs-CRP (by 15%), and elevated GSH (by 52%), GSH/GSSG (by 51%), TAC (by 59%) and CAT (by 15%) (*p* < 0.01). These findings suggest that engagement in chronic intradialytic cardiovascular exercise alters RS, reduces inflammation and improves performance in patients with ESRD.

## 1. Introduction

Kidney diseases are recognized as a tremendous public health burden, with their prevalence exceeding 850 million patients globally [1]. Of note, (CKD), the most common form of kidney disease, affects 8–16% of adult population [2] and is expected to be the 5th leading cause of death worldwide by 2040 [3]. CKD is characterized by lasting damage to kidneys or decreased glomerular filtration rate (GFR) [2] for more than three months [4] and is classified into five stages according to the estimated GFR. According to the United States Renal Data System, the fifth stage, also known as end-stage renal disease (ESRD), led almost 87% of these patients to the most widely used Renal Replacement Therapy (RRT) (i.e., hemodialysis) in 2017 [5]. 

Oxidative stress (OS) and (RS) perturbations are distinctive features of CKD that are evident even in the early stage of the disease [6,7] and increase progressively as the disease progresses [8]. Patients with ESRD, a high percentage of whom are older adults, display increased pro-oxidant activity, which is largely attributed to coexisting comorbidities such as diabetes mellitus (DM), hypertension, metabolic syndrome and atherosclerosis [9,10]. On the other hand, the anti-oxidant capacity of these patients is impaired since reduced levels of vitamin C, D, E, and selenium are received due to (i) dietary restriction of fruits and vegetables (in order to avoid hyperkalemia), (ii) malnutrition and (iii) diminished intestinal absorption, while a deficiency of the redused glutathione (GSH) scavenging system is also evident [9,11,12]. Furthermore, the perturbation of RS in favor of the pro-oxidant state, is further exacerbated by (HD) [9], as the HD process per se contributes to loss of antioxidant molecules and up to a 14-fold increase in the production of reactive oxygen species ROS, following each session [11,13]. Factors that affect the excessive HD-derived (ROS) production include dialyzer membranes, the type of vascular access, the duration of (HD)session, dialysate, anticoagulative and administered drugs [10]. In addition, it has been proposed that HD increases the production of hydrogen peroxide through stimulation of neutrophil burst [14]. Indeed, the development of chronic inflammation is also a hallmark of ESRD, as pro-oxidant molecules and pro-inflammatory mediators interact continuously in kidney diseases, propagating a vicious cycle that results in OS and inflammation [6,15].

Both ROS and inflammation are considered major contributors to endothelial dysfunction, thereby increasing the risk for atherosclerosis and cardiovascular-related morbidity and mortality in these patients [16,17,18,19]. Thus, the establishment of therapeutic interventions that could be efficiently utilized for the reduction of OS and chronic inflammation would be fundamental for the progression of CKD, particularly in patients undergoing HD. Exercise has been shown to drastically alter redox status and intracellular redox-dependent signaling pathways in various populations both acutely and following its systematic implementation [20,21,22,23]. In light of the beneficial effect of exercise training on OS and inflammatory status both in healthy [24,25] and other clinical populations [25], several studies investigated the efficacy of light-to-moderate-intensity intradialytic exercise training in improving the physical status and overall quality of life of the patients, reporting a favorable effect [26,27,28,29,30,31]. In addition, a few investigations have provided indications that engagement to long-term (3 to 4 months) exercise training reduces OS markers in patients with CKD [32,33,34], although acute exercise has been shown to exert a negative effect [30,35].

Therefore, this study aimed at investigating changes in redox status and inflammatory indicators as well as physical performance and overall quality of life in patients with ESRD, following a 6-month intradialytic exercise training intervention.

## 2. Materials and Methods 

### 2.1. Participants and Experimental Design

**Figure 1:**
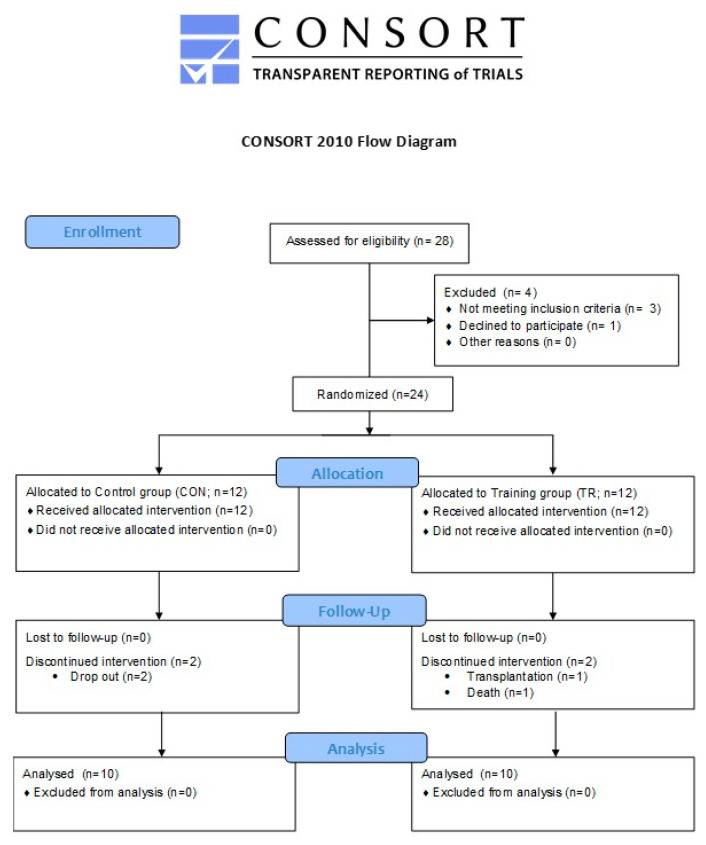
CONSORT flow diagram of the study.

The present study aimed at investigating the efficacy of a 6-month intradialytic cardiovascular training intervention in altering RSand inflammation and improving physical performance in patients with ESRD. All (HD) patients from an outpatient HD unit were informed about the aim and procedures of study, but 28 of them volunteered to participate and were assessed for eligibility. To be included in the study, prospective participants had to meet the following inclusion criteria: (a) participation in chronic therapy for ≥ 12 months prior to the study, (b) underwent 4-h HD sessions 3 times/week with standard bicarbonate dialysis using biocompatible membranes (low flux polysulfone), (c) hemoglobin levels of ≥ 11 g/dL (Erythropoiesis-Stimulating Agents were administered to all patients), (d) no use of antioxidant supplements (i.e., vitamin E, statins or any other medication with antioxidant properties), (e) adequate nourishment (total serum protein 6.8 ± 0.5 g/dL and serum albumin 4.3 ± 0.2 g/dL), (f) no residual renal function, and (g) ability to execute a stationary bike workout. Exclusion criteria included: (a) the presence of an active infectious/inflammatory disease, (b) uncontrolled hypertension and DM, (c) diseases that might interfere with exercise capacity or/and be exacerbated by activity such as ischemic cardiopathy or symptoms related to coronary artery disease, anemia (hemoglobin levels <11 g/dL, Hct < 33%), chronic lung disease and orthopedic disorders, (d) use of steroids, immunosuppressives, and psychotropic agents, and (e) hospitalization within three months prior to the study. Accordingly, of the 28 patients who were initially recruited, 24 were included in the study and 20 finally completed it (1 transplantation, 2 dropouts, 1 death). The CONSORT flow diagram is presented in Figure 1 and participants’ baseline characteristics are demonstrated in Table 1. Participants were informed about the aim of the study, the associated risks and benefits, and provided written informed consent. Procedures were in accordance with the 1975 Declaration of Helsinki and approved by the institutional review board for human research (22/02-06-2010).

A controlled, two-group, repeated measures design was used. Participants were randomly assigned to either a control group (CON, *N* = 10, abstained from exercise training during HD sessions) or a training group (TR, *N* = 10) that participated in a 6-month, supervised, intradialytic training intervention. Before and after the 6-month intervention period, all participants underwent assessment of their anthropometric profile, physical performance level, functional capacity, clinical status and provided a resting blood sample for the determination of OS, inflammatory profile, and antioxidant status. During intervention, CON received only the typical HD sessions, whereas the TR group participated in HD sessions plus in an intradialytic cardiovascular exercise training program.

### 2.2. Exercise Training Program

HD patients in TR participated three times weekly in an intradialytic cardiovascular exercise program performed on a bedside cycle ergometer (Monark Rehab Trainer 881E, Varberg, Sweden) throughout the 6-month intervention period. Each exercise session started 60 min after the initiation of the HD session and consisted of 5 min warm-up, cycling at the desired workload for a self-selected time (depending on each participant’s tolerance) and 5 min cool-down. The exercise intensity was individually adjusted based on the Borg rating scale (6–20 scale) of perceived exertion (RPE) and corresponding at an RPE of 11 (“light”) to 13 (“somewhat hard”). Total workload was progressively increased during the 6-month intervention period, at a self-selected rate (i.e., every month participants were asked to increase the external resistance and the duration of exercise training based on their tolerance). Participants were continuously supervised and monitored by an exercise physiologist and a nephrologist, during each exercise session. Blood pressure, heart rate (HR), RPE and oxygen saturation (SpO_2_) were monitored before, during and after the exercise protocol. Participants were allowed to participate in the exercise program only if they displayed: (i) controlled systolic blood pressure (SBP) and diastolic blood pressure (DBP) and (ii) resting SpO_2_ ≥ 90%. Indications and symptoms for the discontinuation of the exercise regime included: (i) chest pain, (ii) arrhythmias, (iii) dyspnea, (iv) nausea, (v) muscle pain or cramps, (vi) hypotension or hypertension episode and (vii) RPE ≥15 according to Borg’s scale. 

### 2.3. Anthropometric Profile

Body mass was measured to the nearest 0.5 kg and body height to the nearest 0.5 cm by using a beam balance with stadiometer (Beam Balance-Stadiometer, SECA, Vogel & Halke, Hamburg, Germany), as described previously [36]. BMI was calculated as the ratio of weight (kg) to height squared (m^2^) and body fat percentage was determined by the measurement of 7 skinfolds using a Harpenden caliper (John Bull British Indicators Ltd., England) [36]. 

### 2.4. Physical Performance

Participants’ VO_2peak_ was determined during cardiopulmonary exercise stress testing on a stationary cycle ergometer (Monark 834E ergomedic testing bike, Varberg, Sweden) by using a pulmonary gas exchange system (Oxycon Mobile, Yorba Linda, CA, USA), as described [36]. Briefly, the workload was set at 10–20 W over the first 60 s and then was gradually increased by 5–10 W/min, until exhaustion. HR, 12-lead ECG, blood pressure and RPE were continuously monitored during the test and the subsequent recovery period, while a computerized system was utilized to assess oxygen uptake throughout the test via breath-by-breath analysis. The VO_2peak_ was determined as the maximum value on the VO_2_ curve where a plateau was occurred.

### 2.5. Functional Capacity

Participants’ functional capacity was assessed using NSRI walk test and the sit-to-stand test (STS-60), as described [37,38]. Briefly, the NSRI walk test consisted of a 50-m indoor walk (flat ground), stair climbing (climbing up 22 stairs), stair descent (climbing down 22 stairs) and another 50 m walk to the start line. Participants received the instruction to execute the task as fast as possible, and were continuously motivated by the investigators. The total time (in seconds) taken to complete the task was recorded. The STS-60 test involves rising (knees fully extended) and sitting (contact with the chair) back on a chair (standard height) as fast as possible in 60 s, without assistance and represents an indicator of muscle endurance. Participants were instructed to place their hands crossed over the chest and to keep their feet on the ground throughout the testing process. Furthermore, handgrip strength was also measured by using a Jamar hydraulic dynamometer, with participants sitting in standard chair as described [39].

### 2.6. Quality of Life

Self-reported quality of life was assessed using the SF-36 quality of life scoring system [38,40]. It involves 36 questions that are categorized into eight different scales: (i) physical functioning, (ii) role-physical, (iii) bodily pain, (iv) general health, (v) vitality, (vi) social functioning, (vii) role-emotional and (viii) mental health. These scales provide evaluation of two major dimensions: (i) physical health and (ii) mental health and self-evaluation. The average score of the eight scales was considered as the SF-36 final score.

### 2.7. Blood Sampling and Assays

Resting blood samples (~12 mL) were collected pre- (1 day prior to the first training session) and post-training (5 days after the last training session). All samples were collected between 07:00–08:00 (to avoid circadian variations) after an overnight fast, from a forearm vein with subjects in a seated position. A blood portion (~6 mL) was collected in Vacutainer tube, left to clot for 30 min and centrifuged (1500× *g*, 15 min, 4 °C) for serum separation. The supernatant (serum) was transferred to Eppendorf tubes and stored at −80 °C for later analyses of TBARS, PC and hs-CRP. Another blood portion (~6 mL) was collected into tubes containing ethylenediaminetetraacetic acid (EDTA), was immediately centrifuged (1370× *g,* 4 °C, 10 min), and the plasma was collected and dispensed in multiple Eppendorf tubes and stored at −80 °C for later determination of TAC. The remaining plasma erythrocytes were lysed as described [41], and the lysate was later used for CAT, GSH and GSSG determination.

Hs-CRP concentration was quantitatively measured in serum with a latex-enhanced immunoturbidimetric assay, using the Roche Cobas clinical chemistry analyzer, as described [42]. TBARS, PC, TAC, CAT, GSH and GSSG were assayed according to protocols previously described [36,43,44,45,46,47]. Briefly, for the determination of TBARS 100 μL of serum were mixed with 500 μL 35% TCA and 500 μL Tris-HCL (200 mM; pH 7.4) and incubated at room temperature for 10 min. Then, 1 mL of Na_2_SO_4_ (2M)—thiobarbituric acid (55 mM) was added to the solution, and the solution was incubated at 95 °C for 45 min. After incubation, samples were allowed to cool for 5 min, mixed (vortex) after adding 1 mL 70% TCA, centrifuged (15,000× *g*, for 3 min) and the absorbance of the supernatant was then measured at 530 nm. PC were assayed by adding 50 μL of serum to 50 μL of 20% TCA prior to incubation in the ice bath for 15 min and immediately after incubation the solution was centrifuged (15,000× *g*, 4 °C, 5 min). Then, the supernatant was discarded and 500 μL of 2.4-dinitrophenylhydrazine (10 mM in 2.5 N HCL) for the samples or 500 μL of HCL (2.5 N) for the blank was added to the remaining pellet. Thereafter, samples and blank solutions incubated in the dark at room temperature for 1 h with intermittent mixing every 15 min. After incubation, samples and blank solutions centrifuged (15,000× *g*, 4 °C, 5 min), 1 mL of 10% TCA was added to the pellet after discarding the supernatant and centrifuged again at 15,000 *g*, 4 °C for 5 min. The supernatant was then removed, 1 mL of ethanol-ethyl acetate (1:1 v/v) was added to the pellet and centrifuged for 5 min (15,000× *g*, 4 °C). The last procedure repeated two more times, and, thereafter, the supernatant was discarded, the pellet mixed with 1 mL of 5 M urea (pH 2.3) and incubated at 37 °C for 15 min. Finally, the samples and blank solutions centrifuged for 3 min (15,000× *g*, 4 °C) and the absorbance of the supernatant read at 375 nm. For TAC analysis, 20 μL of serum was mixed with 480μL of 10 mM sodium-potassium phosphate (pH 7.4) and 500 μL of 2.2-diphenyl-l picrylhydrazyl (0.1 mM), incubated for 30 min in the dark, at room temperature, and centrifuged (20,000× *g*, for 3 min) before having their absorbance read at 520 nm. For the measurement of CAT activity, 4 μL of red blood cell (RBC) lysate was added to 2991 μL of 67 mM sodium-potassium phosphate buffer (pH 7.4), and the samples were mixed and incubated at 37 °C for 10 min. Subsequently, 5μL of 30% hydrogen peroxide was added in the samples and the change in absorbance was immediately read at 240 nm for 90 s. For GSH determination, 20 μL of RBC lysate was mixed with 660 μL of 67 mM sodium-potassium phosphate buffer (pH 8.0) and 330 μL of 5.5-dithiobis-2-nitrobenzoate (1mM), following treatment with 5% TCA, and then incubated for 45 min in the dark, at room temperature. Immediately after incubation, their absorbance was read at 412 nm. For GSSG determination, RBC lysate was initially treated with 5% TCA (pH 7.0–7.5). Following the addition of 4 μL of 2-vinyl pyridine, samples were incubated at room temperature for 2 h. After incubation, 600 μL of sodium phosphate buffer (143 mM, pH 7.5), 100 μL of NADPH (3 mM), 100 μL of 5.5-dithiobis-2-nitrobenzoate (10 mM) and 194 μL of distilled water were added to samples and incubated at room temperature for 10 min. Thereafter, 1 μL of glutathione reductase was added and the change in absorbance was read at 412 nm for 3 min.

### 2.8. Statistical Analysis

To ensure statistical power in our data analysis, we performed a power analysis prior to the study by using the GPower software (version 3.0.10) and setting an effect size >0.55, a probability error of 0.05 and a power of 0.90 for two groups and two measurement timepoints (pre- and post-training). The analysis indicated that a total sample size of 12 subjects is necessary to detect statistically meaningful differences among groups and timepoints. Accordingly, 24 HD patients were included in the present study and 20 of them finally completed it and included in the analysis. Data are presented as means ± standard deviation (SD). After verification of the normal distribution in our data sets (performed using the Shapiro-Wilk test), parametric tests were applied using the IBM SPSS software (IBM SPSS Statistics 20). A two-way (group × time) repeated measures analysis of variance (ANOVA) with planned contrasts on different time points was used to determine different time point changes on all dependent variables between the control and training groups. Effect sizes (ES) and confidence intervals (CI) were determined according to Hedge’s g method corrected for bias. ES was interpreted as none, small, medium-sized, and large for values 0.00–0.19, 0.20–0.49, 0.50–0.79, and ≥0.8, respectively. Significance was accepted at *p* < 0.05.

## 3. Results

The two groups were comparable at baseline (Table 1), and, as such, an analysis of covariance was not required. In CON 10 out of the 12 patients performed the follow up measurements (two dropouts). In the TR group, one patient underwent transplantation and was discontinued from the study, and one patient passed away. No musculoskeletal injuries or exercise-induced health-related complications were recorded. Consequently, 10 patients completed the study and the follow up assessments (a 81% compliance).

### 3.1. External and Internal Load during the Training Intervention

Participants in TR participated in a 6-month training intervention program incorporating a self-selected intensity and duration. The duration of exercise was significantly increased from 36% during the second month to 46% at the end of training. The external resistance during cycling was gradually increased from 0 to 60.6 W during the 6-month training period. Cycling frequency increased only in 6th month of training from 35 to 41.7 rounds/min. Despite this progressive increase in external load-related parameters, the internal load, i.e., mean systolic and diastolic blood pressure, mean HR, RPE and oxygen saturation remained unaltered throughout the training period. Table 2 presents the average monthly changes in external and internal load characteristics during the training intervention.

### 3.2. Somatometrics

Body mass and body fat percentage decreased by almost 1 kg (*p* = 0.013) and 2% (*p* = 0.002), respectively, in TR, whereas, in CON, both variables remained unaltered (Table 3).

### 3.3. Physical Performance and Quality of Life

Changes in physical performance and quality of life indicators are shown in Table 4. Time to exhaustion (+15%; *p* < 0.001) VO_2peak_ (+15%; *p* < 0.001) and blood lactate concentration (+14%; *p* < 0.001) during the cardiopulmonary exercise stress test increased only in TR.

Similarly, performance was improved by 13% in STS-60 (*p* = 0.001), by 8% in NSRI (*p* < 0.001) and by 1% in handgrip strength testing (*p* = 0.002) only in TR post-training.

Subjective quality of life, as assessed through the SF-36 quality of life scoring system, increased post-training in TR by 4% (*p* = 0.001) and was higher compared to CON (*p* < 0.001). In contrast, no significant changes were observed in SF-36 score in CON throughout the 6-month intervention period.

### 3.4. Inflammation, Oxidative Stress and Antioxidant Status

**Figure 2:**
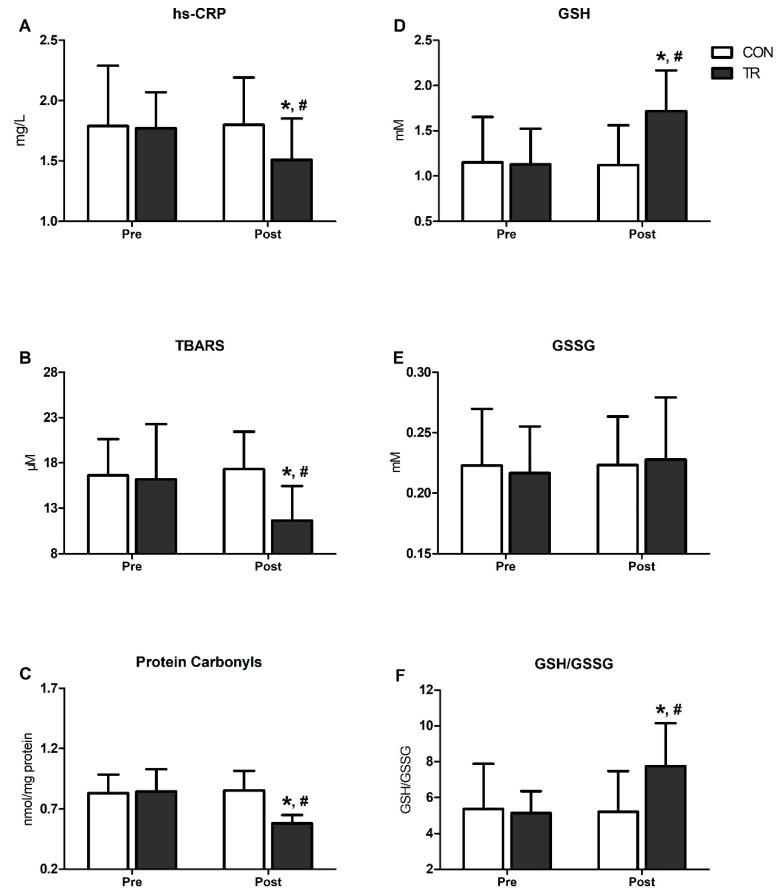
Six-month exercise training intervention reduced oxidative stress and systemic inflammation indices and improved blood redox status. Changes in high-sensitivity-CRP (hs-CRP) (**A**), thiobarbituric acid reactive substances (TBARS) (**B**), proteins carbonyls (**C**), reduced glutathione (GSH) (**D**), oxidized glutathione (GSSG) (**E**) and GSH/GSSG ration (**F**), in training group (TR) and control group (CON) after the 6-month intervention. Data are presented as mean ± standard deviation. * Significant difference with pre-training values (*p* < 0.05); ^#^ Significant difference with the CON group (*p* < 0.05).

Figure 2 presents changes in inflammatory and OS markers in TR and CON. Following the 6-month training intervention, hs-CRP declined by 15% in TR (*p* < 0.001) and was lower compared to CON (*p* < 0.001) (Figure 2A). Likewise, TBARS declined by 28% (*p* < 0.001) and PC by 31% (*p* < 0.001) in TR post-training and were lower than CON (TBARS: CON = 17.3 ± 3.8 vs. TR = 11.6 ± 4.2/PC: CON = 0.85 ± 0.1 vs. TR = 0.58 ± 0.2; *p* < 0.001) (Figure 2B,C). GSH increased by 52% post-training in TR (*p* < 0.001) and was higher compared to CON (CON: 1.12 ± 0.44 vs. TR: 1.72 ± 0.45; *p* < 0.001) (Figure 2D), while no changes were noted for GSSG (Figure 2E). The GSH/GSSG ratio increased by 51% in TR post-training (*p* = 0.001) and was higher than CON (*p* = 0.001) (Figure 2F). All OS indicators (i.e., hs-CRP, TBARS, PC, GSH, GSSG, GSH/GSSG) remained unaltered over time in CON (Figure 2A–F).

**Figure 3:**
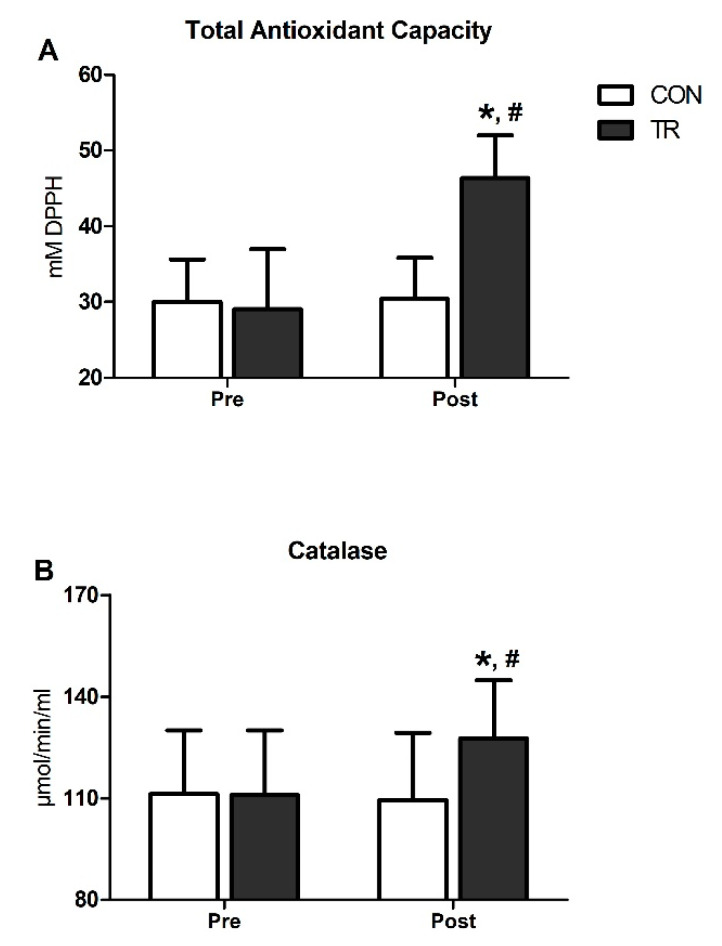
Six-month exercise training intervention upregulated total antioxidant capacity and catalase activity in blood. Changes in total antioxidant capacity (TAC) (**A**) and catalase (CAT) activity (**B**) in TR and CON after the 6-month intervention. Data are presented as mean ± standard deviation. * Significant difference with pre-training values (*p* < 0.05); ^#^ Significant difference with the CON group (*p* < 0.05).

TAC was substantially elevated post-training in TR by 59% (*p* < 0.001) and was higher compared to CON (*p* < 0.001) (Figure 3A). Similarly, CAT increased by 15% in TR post-training (*p* < 0.001) and was also higher than CON (*p* < 0.001) (Figure 3B). Both TAC and CAT remained unchanged in CON throughout the 6-month intervention period (Figure 3A,B).

## 4. Discussion

Exercise training induced favorable changes in RS as well as in the physical and functional performance of patients on HD. Patients who participated in the 6-month exercise training program increased the external load during exercise, whereas internal load markers remained unchanged. Furthermore, TAC, CAT, GSH and GSH/GSSG were increased in patients who participated in the training process, while GSSG remained unaltered and TBARS and PC reduced. No dangerous adverse side effects were reported during exercise training. Only mild elevation of SBP and nausea were observed twice in different patients. 

ESRD patients are suffering from muscle atrophy, reduced endurance and walking capacity which affect the quality of life and mortality [48]. Intradialytic exercise has been introduced as a non-pharmaceutical intervention to counteract the side effects of disease. Previous research papers have shown improvements in physical and functional performance [33,49,50,51]. STS-60 has been theorized as a sensitive functional marker to detect exercise-training improvements in ESRD patients [49,52]. Exercise training results in improvements of 5–14% after 3 months and ~35% after 6 months [49,52]. These changes may reflect improvements in the quality of a patient’s life as well. In agreement with previous studies, an improvement of 13% in STS-60 was observed in this study in response to 6 months of training. In addition, we noticed a small, but significant increase in handgrip strength, despite the fact that we utilized a training intervention that was consisted of lower limb exercise (i.e., cycling). This beneficial effect might be associated with the observed improvement in overall quality of life, suggesting that participants in the TR group became more physically active during the 6-month training intervention (i.e., they were able to perform more physical activities during the day) and consequently the increase in daily activities promoted their handgrip strength. 

Endurance capacity was assessed through VO_2peak_, time to exhaustion [33,50,51] and the NSRI test [33,49,53]. We observed a 15%, 13% and 8% improvement in VO_2peak_, time to exhaustion and NSRI test, respectively. In the same line, Kouidi et al. [50] showed an improvement of 24.8% in VO_2peak_ and 61.4% in time to exhaustion in the same patient population after a 1-year training. On the other hand, Groussard et al. [33] did not observed changes in VO_2peak_ with training despite the fact that performance in the 6-min walk test improved by 11%. NSRI has been reserved as functional performance marker. Sakkas et al. [51], showed a 29% improvement in the NSRI test performance in end-stage kidney patients who participated in an intradialytic exercise training program further corroborating our findings. Differences among studies regarding results are probably related to different characteristics of the training protocol employed (e.g., duration of intervention program, duration of exercise and intensity). The duration of training intervention program seems to be a crucial factor, as longer training interventions have been associated with a more pronounced beneficial effect in a dose-dependent manner [33,49,50,53]. An additional factor that appears to influence the training-induced adaptations is the age of the patients since older patients exhibit more serious functional limitations such as musculoskeletal discomforts and fatigue during the VO_2peak_ determination process [33,54]. 

Following training, we observed a marked reduction in OS markers and enhancement of the antioxidant status indices in TR as compared to CON. Specifically, protein carbonylation and lipid peroxidation were substantially reduced, indicating lower oxidative damage to proteins and lipids, whereas levels of reduced glutathione (GSH) and the GSH/GSSG ratio were elevated, revealing an improvement in blood RS. Furthermore, the endogenous antioxidant defense mechanism was also up-regulated post-training, as evidenced by increased levels of TAC and CAT. Indeed, there is a plethora of studies showing that regular exercise training reduces oxidative damage and up-regulates the activity of antioxidant enzymes, increasing, as such, the resistance to ROS [25]. Beyond the apparent enhancement of TAC and CAT activity that clearly denotes increased antioxidant defense, the up-regulation of GSH observed post-training is of paramount importance. GSH is the most important thiol in cells that acts as a scavenger of free radicals and other reactive oxygen and nitrogen species via enzymatic reactions (mainly via the glutathione peroxidase reaction), mitigating the elevation of OS markers (i.e., PC, TBARS) [55]. It also contributes to the attenuation of GSH/GSSG reduction (redox status) under OS conditions, thus preserving redox status, and regulates redox-sensitive signal transduction pathways responsible for redox and cellular homeostasis [55]. There is a considerable body of evidence reporting that ESRD patients are characterized by extremely low GSH levels and a diminished GSH-scavenging system (i.e., lower GSH levels and reduced GSH peroxidase activity), which leads to exacerbations of OS markers [10,11,17]. Notably, the activity of GSH peroxidase is progressively attenuated as the disease progresses [56]; thus, it seems that OS and GSH deficiency interact in a vicious cycle during the disease, accelerating its progression. Consequently, although acute exercise induces a substantial reduction in blood GSH levels [36], chronic participation to exercise training amplifies GSH [34] and may, therefore, be considered as an efficient, non-pharmaceutical intervention to reduce OS and improve the antioxidant status in ESRD patients. 

To the best of our knowledge, there is currently a limited amount of evidence regarding the RS responses to chronic exercise training in patients with ESRD [24]. In line with our findings, Wilund et al. [32] observed a significant reduction (by 38%) in serum TBARS levels after 4 months of an endurance exercise training program (three intradialytic cycling sessions/week) in patients undergoing haemodialysis, and Groussard et al. [33] reported that F2α-isoprostanes were lower (by 36%) after a 3-month intradialytic cycling exercise intervention in patients with CKD. Furthermore, by implementing low-intensity swimming exercise in the pool for 12 weeks, Pechter et al. [34] showed a significant reduction in lipid peroxidation and enhancement of reduced glutathione levels in patients with moderate renal failure. Therefore, similar to what is evident in healthy [24,25] and other clinical populations [25], chronic participation to exercise training elicits beneficial redox changes in the context of ESRD, though acute exercise either exacerbates or has no impact on OS [30,35]. This exercise-mediated effect can be mechanistically explained by the hormesis theory, according to which regular exercise results in intermittent and transient ROS production and OS, stimulating redox-sensitive signaling pathways that promote protective adaptations against a higher future elevation in ROS levels and molecular damage [57]. 

In terms of systemic inflammation, serum hs-CRP was assessed as a surrogate marker of chronic inflammation in CKD [58,59] and a strong predictor of atherogenic vascular risk and cardiovascular mortality in HD patients [58,59]. In contrast to a previous report where CRP levels remained unaltered in response to a 4-month intradialytic exercise training intervention (cycling) [32], we observed a 15% reduction in hs-CRP in TR, in response to 6 months of exercise training. In fact, engaging in long-term cardiovascular exercise training, particularly of moderate intensity [60], is associated with a reduction in serum CRP levels both in healthy [61] and clinical populations [62,63], and is usually associated with reductions in body fat [61]. Accordingly, the reduction in hs-CRP levels observed in TR was accompanied by a 2% reduction in body fat percentage. In addition, our training program consisted of moderate-intensity bedside cycling (corresponding at a RPE of 11 (“light”) to 13 (“somewhat hard”)), suggesting that even this kind of moderate-intensity, non-weight-bearing exercise training is efficient in alleviating chronic inflammation in patients with ESRD.

However, one limitation in the current study is the fact that habitual physical activity was not monitored during the 6-month intervention period (i.e., neither on HD days nor on non-HD days). This would have enabled us to better understand the impact of our exercise training intervention per se on the dependent variables examined here, as the physical activity performed on HD and non-HD days might have induced and additive effect. Another possible limitation is that dietary macro- and micro-nutrient consumption was not recorded during the experimental period. Given that ESRD patients usually adhere to dietary restriction and/or malnutrition [9,11], a potential disparate dietary profile between our participants might have also interfered with the observed exercise training-induced adaptations.

## 5. Conclusions

Our study demonstrates that even low-to-moderate exercise intensity and duration of training may induce favorable improvements in functional and physical performance markers in ESRD patients. This investigation is the first to report a beneficial effect of low-to-moderate intensity cardiovascular exercise training on OS and systemic inflammation in these patients. Undoubtedly, future studies are required to examine this scenario using a larger sample size, to allow for the extraction of safe conclusions. In any case, ESRD patients should be encouraged to engage in cardiovascular exercise of low-to-moderate intensity as much as possible.

## Figures and Tables

**Table 1:** Participants’ physical and clinical characteristics at baseline.

Variables	Exercise Group (*n* = 10)	Control Group (*n* = 10)
Gender (Female/Male)	2/8	1/9
Age (yr)	52.8 ± 17.1	53 ± 7,6
Body Height (m)	1.71 ± 0.09	1.71 ± 0.1
Body Mass (kg)	72.5 ± 14.6	74.6 ± 9.3
BMI (kg/m^2^)	24.6 ± 3.54	25.5 ± 1.84
Body Fat (%)	27 ± 2.27	27.3 ± 3.54
Dialysis History (months)	88.8 ± 9.9	89.7 ± 10.1
Residual Urea Clearance (ml/min^−1^)	1.32 ± 0,2	1.28 ± 0.3
Intradialytic Weight Gain (kg)	2.66 ± 0.6	2.55 ± 0.6
Dialyzer Clearance of Urea (K_t_/V)	1.33 ± 0.4	1.27 ± 0.3

BMI: Body Mass Index.

**Table 2:** Monthly changes in external and internal load during the 6-month intervention.

Variables	1st Month	2nd Month	3rd Month	4th Month	5th Month	6th Month
Duration of exercise (min)	10.5 ± 0	16.5 ± 1 *	17 ± 2.9 *^,#^	19.8 ± 3.8 *^,#^	22 ± 3.4 *^,#^	24 ± 3.3 *^,#^
Resistance (Watt)	0 ± 0	20 ± 14 *	39.8 ± 24 *^,#^	50.6 ± 28 *^,#^	57 ± 24 *^,#^	60.6 ± 22 *^,#^
Velocity (Rounds/min)	35 ± 0	35 ± 0	35 ± 0	35 ± 0	35 ± 0	41.75 ± 5.4 *^,#^
Mean SBP (mmHg)	148.64 ± 13.8	151.56 ± 12.2	152.62 ± 13.1	155.08 ± 9.1	152.16 ± 11.8	149.64 ± 11.1
Mean DBP (mmHg)	82.48 ± 3.2	81.06 ± 7.6	86.62 ± 7.5	84.98 ± 4.2	85.44 ± 5.1	81.52 ± 7.1
Mean HR (beats/min)	102.72 ± 6.1	104.78 ± 6.9	103.58 ± 6.5	102.5 ± 6.1	103.44 ± 8.7	103.32 ± 8.7
RPE	12.4 ± 0.7	12.1 ± 0.6	11.9 ± 0.4	12.6 ± 0.7	12.2 ± 0.6	11.8 ± 0.3
SpO2 (%)	96.2 ± 1.4	97.3 ± 1.3	96.9 ± 1.5	96.4 ± 1.4	97.5 ± 1.6	96.6 ± 1.5

SBP: Systolic blood pressure; DBP: Diastolic blood pressure; HR: Heart rate; RPE: Rate of perceived exertion. Data are presented as mean ± standard deviation. * Significant difference with the 1st Month, ^#^ Significant difference with the previous month.

**Table 3:** Changes in participants’ body composition.

Variables	Control Pre	Control Post	Experimental Pre	Experimental Post
Body Height (m)	1.71 ± 0.1		1.71 ± 0.09	
Body Mass (kg)	74.6 ± 9.3	74.9 ± 9.13	72.5 ± 14.6	71.5 ± 14 *
BMI (kg/m^2^)	25.5 ± 1.84	25.6 ± 3.19	24.6 ± 3.54	24.2 ± 3.39
Body Fat (%)	27.3 ± 3.54	27.2 ± 2.27	27 ± 2.27	26.8 ± 3.01 *

BMI: Body Mass Index. Data are presented as mean ± standard deviation. * Significant difference with Pre.

**Table 4:** Changes in participants’ physical performance status.

Variables	Control Pre	Control Post	Experimental Pre	Experimental Post
VO_2peak_ (ml/kg/min)	14.8 ± 3.1	14.5 ± 3.03	13.81 ± 3.03	15.9 ± 2.96 *^,#^
Time to Exhaustion (min)	9.74 ± 1.7	9.64 ± 1.69	9.8 ± 2.69	11.3 ± 2.16 *^,#^
Resting HR (beats/min)	79 ± 9.9	79.7 ± 6.9	78.4 ± 13.2	77 ± 11.4
Peak HR (beats/min)	122.9 ± 17.6	122.3 ± 16.9	121.5 ± 17.3	122.4 ± 16.1
Resting Lactate (mM)	0.98 ± 0.26	1 ± 0.19	0.99 ± 0.2	0.97 ± 0.17
Peak Lactate (mM)	6.1 ± 1.1	6.02 ± 1.06	6.2 ± 1.6	7.07 ± 1.35 *^,#^
STS-60 (reps in 60 s)	33 ± 7.6	32.25 ± 7	33.83 ± 7.2	38.08 ± 6.3 *^,#^
NSRI test (s)	51 ± 8.78	49.9 ± 7.86	49.8 ± 10.6	53.7 ± 10.5 *^,#^
Handgrip Strength (kg)	24.7 ± 9.65	23 ± 10.2	23.4 ± 10.5	23.67 ± 10.16 *^,#^
SF-36	17.5 ± 3.5	17.6 ± 2.6	17.2 ± 3.3	15.3 ± 3.1 *^,#^

HR: Heart rate; STS-60: Sit-to-Stand – 60s test; NSRI: North Staffordshire Royal Infirmary; SF-36: Short form-36 quality of life scoring system. Data are presented as mean ± standard deviation. * Significant difference with pre training intervention values; ^#^ Significant difference with the control group.

## Abbreviations

BMI	body mass index
CAT	catalase
CI	confidence intervals
CKD	chronic kidney disease
CON	control group
CVD	cardiovascular disease
DBP	diastolic blood pressure
DM	diabetes mellitus
EDTA	ethylene diamine tetraacetic acid
ES	effect sizes
ESRD	end-stage renal disease
GFR	glomerular filtration rate
GSH	redused glutathione
GSSG	oxidized glutathione
HD	hemodialysis
HR	heart rate
hs-CRP	high-sensitivity C-reactive protein
NSRI	north staffordshire royal infirmary
OS	oxidative stress
PC	protein carbonyls
PEW	protein wasting energy
ROS	reactive oxygen species
RPE	rate of perceived exertion
RRT	renal replacement therapy
RS	redox status
SaO_2_	oxygen saturation
SBP	systolic blood pressure
SF-36	short form-36
STS-60	sit to stand-60
TAC	total antioxidant capacity
TBARS	thiobarbituric acid reactive substances
TCA	trichloro acetic acid
TR	training group
VO_2peak_	peak oxygen consumption
